# Mind wandering and education: from the classroom to online learning

**DOI:** 10.3389/fpsyg.2013.00495

**Published:** 2013-08-01

**Authors:** Karl K. Szpunar, Samuel T. Moulton, Daniel L. Schacter

**Affiliations:** ^1^Department of Psychology, Harvard UniversityCambridge, MA, USA; ^2^Harvard Initiative for Learning and Teaching, Harvard UniversityCambridge, MA, USA

**Keywords:** mind wandering, attention, educational psychology, learning, teaching, online learning

## Abstract

In recent years, cognitive and educational psychologists have become interested in applying principles of cognitive psychology to education. Here, we discuss the importance of understanding the nature and occurrence of mind wandering in the context of classroom and online lectures. In reviewing the relevant literature, we begin by considering early studies that provide important clues about student attentiveness via dependent measures such as physical markers of inattention, note taking, and retention. We then provide a broad overview of studies that have directly measured mind wandering in the classroom and online learning environments. Finally, we conclude by discussing interventions that might be effective at curbing the occurrence of mind wandering in educational settings, and consider various avenues of future research that we believe can shed light on this well-known but little studied phenomenon.

During the past decade, there has been impressive growth in research concerning the cognitive and neural bases of mind wandering, including a rapid expansion of experimental procedures that have rendered the phenomenon tractable for experimental studies, a growing body of reliable findings, and a number of theoretical proposals aimed to account for the phenomena of interest (for reviews, see Smallwood and Schooler, 2006; Smallwood, 2013). During the same time period, there has been a similarly impressive increase in the application of findings and ideas from cognitive psychology to understanding learning and retention in educational contexts (for recent reviews, see Roediger and Karpicke, 2006; Bjork et al., 2013; Dunlosky et al., 2013). It seems clear that these two domains of research should be highly relevant to one another, because mind wandering and related attention failures are widely recognized to be common in the traditional classroom setting (e.g., Johnstone and Percival, 1976; Bligh, 2000; Bunce et al., 2010) as well as in online education (e.g., Koller, 2011; Khan, 2012). Perhaps surprisingly, there has been relatively little research linking the two domains; indeed, only a few years ago, Smallwood et al. (2007) characterized mind wandering as an “underrecognized” influence in educational settings and provided a useful discussion of experimental results and conceptual/theoretical considerations relevant to linking the two domains.

In the past couple of years, systematic research has begun to emerge that focuses on the incidence and nature of mind wandering in both traditional classrooms as well as online learning environments. The primary purpose of the present article is to provide a focused review and discussion of recent research, as well as some lesser known older studies that examine the occurrence and consequences of mind wandering during both classroom and online lectures. In addition, we consider possible interventions for reducing the occurrence of mind wandering in educational settings and conclude by discussing potentially fruitful directions for future research.

## Mind wandering during classroom instruction

Within educational settings, the occurrence of mind wandering is perhaps most readily observable within the context of classroom instruction. Indeed, educators have long been concerned about the possible negative impact of mind wandering on student learning (Brown, 1927; Lloyd, 1968). It is important to note, however, that few studies have directly measured mind wandering in the classroom. Instead, early research made use of measures such as physical markers of inattention, note taking, and retention. Data emerging from these early studies revealed important clues about the nature of student attentiveness over extended periods of study that have helped to guide more recent research on mind wandering in the classroom. In this section, we review and evaluate the basic findings emerging from these early studies, discuss the possible relation of these findings to mind wandering, and highlight direct attempts to measure mind wandering in the classroom. In addition, we assess the influence of possible interventions for reducing the occurrence of student mind wandering.

### Observational approaches

In what is often cited as a classic example of student attentiveness in the classroom, Johnstone and Percival (1976) asked observers to make note of physical signs of inattention, such as diversions in gaze, as students sat through chemistry lectures. The authors found that initial breaks in attention occurred after approximately 10–18 min of class time, and that the frequency of breaks in attention rose to a level of every 3–4 min toward the end of lectures. Indeed, the notion that student attentiveness decreases as a function of time spent in the classroom has strongly influenced research in this area. Nonetheless, it is important to note that physical markers of inattention should be interpreted cautiously (Wilson and Korn, 2007). For instance, students who have momentarily directed their gaze away from the lecturer may still be listening to the lecturer, and not necessarily mind wandering; conversely, a focused gaze does not necessarily indicate a focused mind. Importantly, recent studies have drawn stronger links between physical markers of inattention and mind wandering. For example, Smilek et al. (2010) recently assessed the relation of blinking to mind wandering during a reading task. In that study, students were asked to indicate whether or not they were paying attention to the text in response to a series of auditory tones. The authors found that blinking was more likely to precede moments of inattention than attention, and suggested that blinking might facilitate the decoupling of attention from the immediate environment during instances of mind wandering. Moving forward, additional research is needed to demonstrate how physical markers of inattention relate to the occurrence of mind wandering in the classroom (for relevant discussion, see Bligh, 2000; Rosengrant et al., 2011).

### Note taking and retention

Various attempts have been made to circumscribe the difficulties associated with inferring student attentiveness via direct observation. For instance, some researchers have focused on note taking. Although note-taking behavior does not necessarily correlate with comprehension (e.g., McClendon, 1958), reductions in note taking over time may indicate inattention on the part of students. Unfortunately, the conclusions that can be drawn on the basis of relevant data are equivocal. For instance, Maddox and Hoole (1975) and Scerbo et al. (1992) examined the percentage of ideal notes (notes deemed important by the experimenter) that students recorded during lectures (for further discussion on research approaches to note taking, see Aiken et al., 1975). Maddox and Hoole (1975) found no decline in note taking across five 10-min intervals of a geography lecture—44, 54, 50, 52, and 55% of ideal notes. Conversely, Scerbo et al. (1992) observed a steep decline in note taking across three 12-min intervals of a psychology lecture—97, 67, and 50% of ideal notes (see also Hartley and Cameron, 1967; Locke, 1977). One possibility for this discrepancy may be related to factors such as student interest. For instance, students in the geography class (51%) took significantly fewer notes across the entire lecture than students in the psychology class (71%), and high levels of initial note taking may be necessary to observe subsequent declines over time. Moreover, additional studies are needed to demonstrate the extent to which inattention and declines in note taking co-occur. Along these lines, Lindquist and McLean (2011) recently demonstrated that frequent bouts of mind wandering—as measured by direct probes of attention—were associated with lower subjective ratings of note taking. Whether this observation extends beyond subjective reports of note taking to actual note taking behavior remains to be tested.

Alternatively, various researchers have looked to measures of retention as proxies for student attentiveness in the classroom. Specifically, if students are less likely to pay attention to the latter portion of a lecture, then information presented toward the end of the lecture should not be retained as well as information presented in earlier portions of the lecture. Again, the evidence is somewhat mixed. While some studies have found reduced memory for information presented at the end of lectures (Burns, 1985), others have not (Thomas, 1972; Scerbo et al., 1992 for additional discussion, see McLeish, 1968). One possibility for this unreliable pattern of data is that the critical test is commonly presented immediately after the lecture. This design feature may allow students to rehearse information from the final portion of the lecture until the test is administered (Glanzer and Kunitz, 1966). In order to more accurately assess what information students have integrated into their knowledge base, additional studies ensuring that students express their understanding of lecture content on the sole basis of long-term memory are needed. In addition to possible primacy and recency effects (e.g., Jersild, 1929; Ehrensberger, 1945), future studies might also consider the possible influence of other factors that might moderate attention over extended periods of time, such as the distinctiveness or relation of materials to one another across an entire lecture.

Although little is known about the relation of the occurrence of mind wandering and retention of lecture content, Lindquist and McLean (2011) showed that the frequency of mind wandering in response to direct probes of attention during one lecture was negatively correlated with retention of course material on an exam taken several weeks later. Moving forward, it will be important to more closely investigate the extent to which mind wandering accounts for both the immediate and long-term retention of specific materials from lectures.

### Direct probes of attention and mind wandering

We now discuss in more detail studies that have used direct probes of student attention and mind wandering. These studies are important because they provide a more accurate depiction of the extent to which students are actually mind wandering in educational contexts. In one of the initial studies of this sort, Cameron and Giuntoli (1972) randomly interrupted college lectures with a bell and asked students various questions about the content of their conscious mind, including whether or not they were listening to the speaker, and, if so, whether their listening was “a superficial kind of listening accompanied by frequent distractions,” “a close following of the speaker's train of thought,” or a kind of listening in which they felt that they were “actively meeting the speaker's mind.” Depending on how one classifies students' responses, the results revealed that only between 40 and 46% of students were paying “good attention” to the lecturer or lecture content at any given moment. Using a similar method of consciousness sampling in undergraduate and graduate classrooms, Schoen (1970) estimated attention during lectures at only 67%, whereas attention during discussion was estimated at 75% (see also Geerligs, 1995) and attention during problem solving was at 83%.

Stuart and Rutherford (1978) asked medical students in twelve 50-min hematology lectures to indicate the extent to which they were paying attention using a 9-point scale (1 = not concentrating at all; 9 = maximum concentration). A buzzer that was audible to students sounded the attention probes at 5-min intervals. The authors found that students, on the whole, never indicated more than an “average level of concentration” throughout the lecture. Interestingly, the authors also found that student attention peaked around 10–15 min into the lecture, but that their attention waned considerably thereafter (see also, Johnstone and Percival, 1976; for possible alternative interpretations, see Wilson and Korn, 2007).

In a more recent study, Lindquist and McLean (2011) more directly assessed the occurrence of mind wandering during lectures. Specifically, the authors asked students in three 50-min psychology lectures to report on the occurrence of task unrelated thoughts in response to auditory attention probes that were sounded on five separate occasions—8, 15, 25, 34, and 40 min. Across the entire lecture, task unrelated thoughts were reported in response to ~33% of the attention probes. Moreover, the authors found that task unrelated thoughts were more likely to be reported at the end of the lecture (44%) than the beginning of the lecture (25%). As discussed earlier, Lindquist and McLean also demonstrated a negative impact of mind wandering on note taking and retention. We will revisit this important feature of the authors' data in the context of learning from online lectures, where researchers have greater control over study materials.

Other researchers have used experience sampling paradigms to estimate student attention in everyday life, and such results help contextualize the findings from classroom environments. Unsworth et al. (2012) asked students to record in a diary their attentional failures during everyday life, and found that the most frequent failures were distraction while studying and mind wandering in class; moreover, 76% of the reported lapses of attention—distraction, mind wandering, or absent-mindedness—occurred in classroom or study situations. Kane et al. (2007) asked undergraduates to report whether their minds were wandering at random times during the day. On the average, students' minds were wandering 30% of the time (see also, Hurlburt, 1979). Furthermore, mind wandering increased when students reported they were tired, stressed, and in boring or unpleasant activities. McVay et al. (2009) measured mind wandering in the everyday lives of college students, who similarly reported mind wandering on 30% of the samples. Here again, mind wandering was more frequent when students reported feeling tired or anxious, or when they rated the current activity as stressful or boring. Interestingly, mind wandering was also less frequent when participants reported being happy (see also, Killingsworth and Gilbert, 2010), good at the current activity (see also Moneta and Csikszentmihalyi, 1996), liking the current activity, or rating it as important.

It is important to note that assessments of mind wandering in different contexts are complicated in several important ways. For instance, educational activities such as sitting through a lecture and studying for an exam typically require sustained attentional focus, whereas non-educational everyday activities such as eating breakfast or checking the mail do not necessarily require an individual's undivided attention. Moreover, the consequences of mind wandering also depend on context: The cost of attentional failures during the attention-demanding tasks of education are almost certainly greater than the cost of attentional failures during highly rehearsed, largely automatic tasks of everyday life. As a result, mental experiences such as thinking about a recent or upcoming personal experience may be classified as mind wandering in one context but not the other, and may impact performance in one context but not the other.

In sum, studies making use of direct measures of student attention in educational settings have demonstrated that students frequently report lapses of attention and mind wandering in the classroom, mind wandering appears to increase as a function of time spent in class, and mind wandering may be especially prevalent in educational, as compared to non-educational, settings. Taken together, studies of student mind wandering in the classroom highlight the need for evidence-based research that considers the manner in which classroom instruction is structured, and what interventions might be effective for holding student interest and attention.

### Classroom interventions

Educational guidelines commonly urge teachers to intersperse their lectures with tasks that can help to re-focus student attention (e.g., Myers and Jones, 1993; Middendorf and Kalish, 1996; see also, Olmsted, 1999). Unfortunately, only a few attempts have been made to test the effectiveness of such techniques, and the data are often difficult to interpret.

For instance, Burke and Ray (2008) tested the efficacy of four active learning interventions (student-generated questions, guided reciprocal peer questioning, truth statements, and think-pair-share) across four instructional theory lectures. Each lecture was devoted to testing one of the four interventions, with the intervention occurring halfway through lecture. During each lecture, students were asked to rate their concentration levels on five separate occasions using a 4-point rating scale (1 = not concentrating at all; 4 = fully concentrating), including once at the start of class and once after the intervention. Although the authors demonstrated enhanced levels of concentration following some interventions (student-generated questions) and not others (truth statements), there was no baseline condition against which these effects could be evaluated. Additionally, the order in which students encountered the interventions was not counterbalanced (see also, Young et al., 2009). As a result, it is difficult to know for certain how effective the various interventions were in focusing the attention of students.

More recently, Bunce et al. (2010) asked students in three 50-min chemistry lectures to use clicker technology to indicate whenever their attention to lecture content had been drawn away by various distractions (e.g., texting, completing homework from other courses). In addition, the authors noted various pedagogical techniques used by the instructors of these lectures (e.g., lecturing, quizzing, demonstrations). Although the implementation of the pedagogical techniques was not experimentally manipulated, the authors found that bouts of distraction during lectures were reduced following quizzes and demonstrations. It is also important to note that attentiveness to lecture content was measured via self-reports of distraction that are potentially limited because students are often unaware that they are mind wandering (Smallwood and Schooler, 2006; but see recent neuroimaging data suggesting common neural correlates for subjective and objective reports of mind wandering; Smallwood et al., 2008). Nonetheless, the results of this study are informative, and additional studies that carefully manipulate that frequency and timing of active learning interventions in the classroom, and that assess distraction and mind wandering in a more direct or objective manner, will be of considerable importance.

Next, we delve into the world of online education, and consider the limitations that mind wandering places on effective learning of lecture videos. As discussed below, the advent of online learning is of great interest in its own right in light of its recent prominence on the educational scene. Moreover, using online lectures as target materials has made it possible to study the occurrence of mind wandering during lectures, and explore possible interventions for reducing mind wandering, with tighter experimental control than is typically available in the classroom.

## Mind wandering during online lectures

The studies discussed in the preceding section indicate that mind wandering occurs frequently in the classroom and while studying. As noted earlier, in recent years there has been rapidly growing interest in online education. While online education has existed in some form for nearly as long as the Internet has been around, the emergence of such online platforms as Coursera and edX, which are composed of leading research universities, has led to a dramatic increase in the number of students participating in the entity known as a MOOC or massive open online course. The primary form of instruction in a MOOC is a videorecorded lecture delivered online. Given the frequent occurrence of mind wandering in the traditional classroom, an important question concerns whether mind wandering occurs to a similar, greater, or lesser extent in online settings. While there is very little systematic research on the topic, relevant data have been provided by two recent studies in which participants viewed videorecorded classroom lectures that to some degree resemble those used in online courses. Importantly, by mimicking the online experience in the laboratory, researchers have been able to bring the lecture learning experience, measures of the occurrence of mind wandering during lectures, and tests of possible interventions to ward off mind wandering during lectures under greater experimental control.

Risko et al. (2012) reported two experiments in which students watched videorecorded lectures—alone in Experiment 1, and with other students in a classroom setting in Experiment 2. Risko and colleagues showed participants one of three 1-h lectures on different topics (psychology, economics, or classics). In Experiment 1, 60 undergraduates watched the lectures and were probed at four different times into a lecture—5, 25, 40, and 55 min. During each probe, students were asked if they were mind wandering at that moment. Overall, participants indicated that they were wandering in response to 43% of the probes, with significantly more mind wandering observed in response to the two probes given during the second half of the lecture (52%) than to those given during the first half (35%). The increase in mind wandering across the lecture was associated with poorer performance on a test of lecture material given shortly after the lecture: students responded correctly to 57% of questions concerning the second half of the lecture, compared with 71% correct responses to questions concerning the first half of the lecture. Further, there was a significant negative correlation between test performance and mind wandering (*r* = −0.32): individuals who performed more poorly on the test reported more mind wandering. Experiment 2 yielded a highly similar pattern of results: students reported mind wandering in response to 39% of probes, reports of mind wandering increased significantly from the first half of the lecture (30%) to the second (49%), and mind wandering during the second half of the lecture was associated with significantly poorer test performance compared with the first half of the lecture (for similar results, see Risko et al., 2013).

The incidence of mind wandering during videorecorded lectures was notably high—at least as high as the rate of mind wandering during classroom lectures reported by Lindquist and McLean (2011). One possible contributing factor is the 1-h length of the videorecorded lectures used by Risko et al. (2012). Some advocates of online education, such as Salman Khan, founder of the highly successful Khan Academy, and Daphne Koller, co-founder of Coursera at Stanford University, have argued that online lectures should be brief—as short as 10 min—in part because of concerns raised by earlier studies of classroom lectures, as discussed above, showing that individuals cannot sustain attention for longer periods of time (Koller, 2011; Khan, 2012; for possible limitations associated with this view, see Wilson and Korn, 2007). Thus, it is possible that mind wandering would occur much less often during videorecorded lectures that are considerably shorter than the 1-h lectures used in the Risko et al. (2012) study.

Szpunar et al. (2013) addressed this issue in a study that used a 21-min videorecorded lecture. This study also examined the critical and as yet unaddressed question of whether it is possible to reduce mind wandering during an online lecture. Szpunar et al. (2013) addressed the question by interpolating brief tests within the lecture. Previous research using materials such as word lists, face-name pairs, and prose passages has shown that interpolating brief tests at regular intervals between lists of stimuli can help to improve retention of materials from the end of extended study sequences (see Szpunar et al., 2008; Weinstein et al., 2011; Wissman et al., 2011).

Szpunar et al. (2013) reported two experiments in which participants watched a 21-min videorecorded statistics lecture (results of the two experiments were very similar; here we focus on Experiment 2). The lecture was divided into four segments of equal length. Prior to the lecture, all participants were instructed that they might or might not be tested after each segment, and that they would also receive a final test at the conclusion of the lecture. Participants were encouraged to take notes during the lecture. After each lecture segment, all participants completed arithmetic problems unrelated to the lecture for about a minute. However, there were three different groups, which were defined by what the participants did next: the *tested* group received brief tests on each segment that took about 2 min each; the *non-tested* group did not receive a test until after the final segment, and continued to work on arithmetic problems for an additional 2 min for each of the segments preceding the final segment; and the *re-study* group did not receive a test until after the final segment, and was shown, but not tested on, the same material as the tested group for 2 min for each of the segments preceding the final segment. At random times during the lectures, participants in all groups were probed about whether they were paying attention to the lecture or mind wandering off to other topics.

Participants in the non-tested and re-study groups indicated that they were mind wandering in response to about 40% of the probes, but the incidence of mind wandering was cut in to half, to about 20%, in the tested group. Moreover, participants in the tested group took significantly more notes during the lectures (three times as many), and retained significantly more information from the final segment of the lecture, than did than participants in the other two groups, who performed similarly. Participants in the tested group were also less anxious about a final test that followed the lecture and performed significantly better on that final test than those in the other groups. These results indicate that part of the value of testing comes from encouraging people to sustain attention to a lecture in a way that discourages task-irrelevant activities such as mind wandering and encourages task-relevant activities such as note taking.

Taken together, the results of the studies by Risko et al. (2012, 2013) and Szpunar et al. (2013) suggest that mind wandering occurs frequently during the viewing of online lectures regardless of lecture length: both studies found evidence of mind wandering in response to about 40% of probes in non-tested conditions, even though the lectures used by Risko et al. were three times as long as those used by Szpunar et al. We think that these estimates of mind wandering are probably conservative when one considers the conditions that characterize online learning in everyday life: many students may view online lectures under conditions conducive to mind wandering and distraction, such as at home or in dorm rooms that are full of potentially attention-diverting material such as friends, television, Facebook, e-mail, and the like (for further discussion, see Risko et al., 2013).

It is encouraging that interpolated testing can dramatically reduce the incidence of mind wandering, and increase the incidence of task-relevant activities such as note taking. Such findings provide some confirmation for those practitioners of online learning who are already incorporating interpolated testing into their online lectures. Nonetheless, the results reported by Szpunar et al. (2013) must be treated with some caution, both because they were obtained only with a single lecture on a single topic (i.e., statistics), hence raising the question of whether the beneficial effects of testing can be observed across lectures on a variety of topics, and also because it is unclear whether the benefits of testing will persist across multiple lectures. For example, it is possible that students become less responsive to interpolated testing as an online course goes on (Dyson, 2008). Given the paucity of data available concerning processes and variables that affect learning from online lectures, these and related questions will be important to address in future studies.

## Concluding comments

In sum, early research using proxies of student attention such as physical manifestations of inattentiveness, note taking, and retention, along with more recent studies that more directly probe for instances of mind wandering, highlight the prevalence of attentional lapses and mind wandering in the classroom and during online learning. To some extent, student mind wandering reflects a larger reality of human mental life: just as our minds wander frequently in everyday life, they also wander frequently in educational settings. But mind wandering is particularly relevant to education for two reasons. First, on theoretical and empirical grounds, there is good reason to think that mind wandering is particularly prevalent in educational settings. Online or in the classroom, instruction and studying demand unusually sustained periods of student attention in the presence of unusually salient distractors. In everyday life, one is not typically expected to listen attentively to an hour-long presentation twice a day in a large room full of one's peers, or read large amounts of challenging literature on one's own time instead of socializing or browsing the internet. The attentional demands of lecturing or studying differ from the attentional demands of commuting, cooking, or conversing with colleagues. And as the studies we have summarized (e.g., Unsworth et al., 2012) suggest, mind wandering does seem to occur more frequently during instruction and studying than other activities.

Secondly, mind wandering is particularly relevant to education because learning depends critically on attention in ways that other activities do not. Indeed, engaging student attention is often considered an essential feature of education. In a recent survey of nearly 200 Harvard faculty (Advancing the science, 2013), they were asked to complete the following sentence: “For me, an essential of good learning or teaching is _________.” By far, the most common response was “engagement,” and we suspect students, teachers, and educators of all stripes would agree about the central importance of student engagement. Learning experiences—whether they occur in the classroom, library, dining hall, or online—are intended to engage student attention. And for good reason: If a student does not attend consciously to instruction due to an episode of mind wandering, then that student's learning is surely diminished, both for the content not initially encoded and any subsequent content that depends on this initial learning. Thus, because learning is the goal of instruction and studying—and because learning depends on attention—mind wandering presents a particular challenge to education.

What can students or instructors do to reduce unwanted mind wandering during instruction? As we outlined above, there is some preliminary evidence that interspersing periods of instruction with low-stakes quizzing can promote student attention. We also noted earlier that instructors are commonly encouraged to mix up the content of their lectures (Middendorf and Kalish, 1996). In fact, cognitive psychologists have demonstrated that interleaving the presentation of various interrelated topics as opposed to dealing with each one in turn can help students to avoid confusing related concepts (e.g., Rohrer, 2012). Whether these approaches are effective because frequent changes of topic or brief exposures to any single topic—as compared to prolonged exposure to a single topic—help to sustain students' attention remains an open question for future research. Indeed, education researchers and psychologists have not satisfactorily explored how pedagogy affects mind wandering. To give another example, a considerable amount of research has demonstrated that spacing study over multiple learning sessions as opposed to massing (or cramming) study into a single learning session is a more effective means of ensuring long-term retention of classroom materials (Cepeda et al., 2006; Pashler et al., 2007; Dunlosky et al., 2013) One interesting question for future research may be to examine the extent to which spaced, as compared to massed, study sessions are resistant to bouts of mind wandering and inattention. Given the relative ease of thought sampling methodology and relative importance of student attentiveness, we encourage researchers to expand the empirical literature.

To better understand the causes of and countermeasures against student mind wandering, it is perhaps worthwhile to consider contrasting scenarios. First, how does the experience of attending a lecture differ from the experience of attending other events as an audience member? Indeed, students face attentional requirements during instruction very similar to those of other audiences who passively watch extended presentations. In attending a lecture instead of a movie screening, musical performance, or theatrical performance, however, many of the situational interventions designed to avoid distraction are absent: smartphones and laptop use is allowed (or even encouraged) not banned, lighting is flat instead of focused, the audience whispers, enters, or exits with relative freedom, the stage is bare instead of carefully designed, the presented visuals are often textual, static, or basic instead of graphic, dynamic, and complex, and the audio narration is more likely to be monotonous than lively. For these reasons and others, the conscious experience of watching a 2-h movie is likely very different from that of attending a 2-h lecture.

Other experiments, imagined or real, might be equally revealing. For example, why does the conscious experience of a lecturer differ so greatly from those of the lectured? While students listening to a lecturer wander in their thoughts about a third of the time, the lecturer is typically able to maintain her attention during the same time period and in the same physical space. Why does this simple shift of perspective make such a difference? Might it be the distinction between activity and passivity (e.g., active engagement via intermittent quizzing seems to help), or the asymmetry of the social dynamics between student and instructor? Indeed, recent studies of online learning suggest that asking students to take the perspective of the instructor and teach concepts to virtual students helps to improve retention of course content (Chase et al., 2009). Furthermore, perhaps the dramatically different perspective between the lecturer and the lectured furthers the problem of student mind wandering: If the lecture is extremely engaging for the lecturer but less so for students, then this difference of perspective might discourage lecturers from better designing instruction to engage student attention.

Finally, although we have focused considerable attention on the possible pitfalls of mind wandering during classroom and online learning, there also exists the possibility that mind wandering may in some instances benefit the learner. For instance, Baird et al. (2012) recently demonstrated that the occurrence of mind wandering during a period of incubation was positively correlated with the ability of students to generate solutions to problems designed to test creativity. Under what circumstances might mind wandering benefit classroom or online learning? Do individual differences in the characteristics of mind wandering episodes or propensity to engage in mind wandering predict whether mind wandering might help or hinder learning? Studies designed to answer these and similar questions might not only result in concrete recommendations to students and instruction, but might also uncover new insights into mind wandering, attention, and psychology.

### Conflict of interest statement

The authors declare that the research was conducted in the absence of any commercial or financial relationships that could be construed as a potential conflict of interest.
